# Synthesis, Structure, Hydrodynamics and Thermoresponsiveness of Graft Copolymer with Aromatic Polyester Backbone at Poly(2-isopropyl-2-oxazoline) Side Chains

**DOI:** 10.3390/polym12112643

**Published:** 2020-11-10

**Authors:** Elena Tarabukina, Emil Fatullaev, Anna Krasova, Mikhail Kurlykin, Andrey Tenkovtsev, Sergei S. Sheiko, Alexander Filippov

**Affiliations:** 1 Institute of Macromolecular Compounds, Russian Academy of Sciences, 199004 Saint-Petersburg, Russia; krasova_anna@bk.ru (A.K.); mike_x@mail.ru (M.K.); tenkovtsev@yandex.ru (A.T.); sergei@email.unc.edu (S.S.S.); afil@imc.macro.ru (A.F.); 2 School of Photonics, St. Petersburg National Research University of Information Technologies, Mechanics and Optics, 199004 Saint-Petersburg, Russia; ximik53@yandex.ru; 3 Department of Chemistry, University of North Carolina, Chapel Hill, NC 27599-3290, USA

**Keywords:** graft copolymers, thermoresponsive polymers, aromatic polyester, poly(2-isopropyl-2-oxazoline), thermoresponsiveness, microphase separation, dilute solutions, light scattering

## Abstract

New thermoresponsive graft copolymers with an aromatic polyester backbone and poly(2-isopropyl-2-oxazoline) (PiPrOx) side chains are synthesized and characterized by NMR and GPC. The grafting density of side chains is 0.49. The molar masses of the graft-copolymer, its backbone, side chains, and the modeling poly-2-isopropyl-2-oxaziline are 74,000, 19,000, 4300, and 16,600 g·mol^−1^, respectively. Their conformational properties in nitropropane as well as thermoresponsiveness in aqueous solutions are studied and compared with that of free side chains, i.e., linear PiPrOx with a hydrophobic terminal group. In nitropropane, the graft-copolymer adopts conformation of a 13-arm star with a core of a collapsed main chain and a PiPrOx corona. Similarly, a linear PiPrOx chain protects its bulky terminal group by wrapping around it in a selective solvent. In aqueous solutions at low temperatures, graft copolymers form aggregates due to interaction of hydrophobic backbones, which contrasts to molecular solutions of the model linear PiPrOx. The lower critical solution temperature (LCST) for the graft copolymer is around 20 °C. The phase separation temperatures of the copolymer solution were lower than that of the linear chain counterpart, decreasing with concentration for both polymers.

## 1. Introduction

Physicochemical properties of molecular brushes (grafted copolymers) in solution and in the bulk strongly depend on architectural parameters, such as degree of polymerization of the main chain, length and grafting density of the side chains [1,2,3,4,5]. Excluded volume repulsion of densely grafted side chains determines the conformation of molecular brushes in good and Θ-solvents by enhancing backbone stiffness characterized by the Kuhn length *A*. The length scale of this effect depends primarily on the side chains length *L_s_* and their grafting density *z* [5,6,7,8]. In addition to architecture, solution properties of grafted copolymers depend on the interaction of chemically dissimilar side chains and backbone [9,10,11]. Of particular interest are water soluble grafted copolymers constructed from synthetic and natural components such as cellulose and chitosan, which are used in many biomedical applications [12,13,14,15,16].

One of the ways to synthesize graft copolymers is the so-called polymerization-polycondensation reaction, whereby polycondensation yields the main chain, followed by grafting from polymerization of side chains [17,18,19,20]. For such high molecular weight compounds, most solvents are selective. Therefore, the thermodynamic quality of the solvent with respect to the backbone and side chains plays an important role. The solution behavior of amphiphilic graft copolymers has been studied for aromatic polyimides grafted with vinyl polymers such as polystyrene, polymethylmethacrylate, poly-tert-butyl acrylates, their copolymers with polymethacrylic acid [21,22,23,24,25], and enzymatically synthesized polyesters grafted with hydrophilic and hydrophobic side chains [26]. It is shown that copolymers with a low grafting density tend to form supramolecular structures due to aggregation of the polyimide backbones. Higher grafting density and longer side chains favors molecular solutions with conformations varied from spherical in theta solvents to rod-like in good solvents. Changing the degree of grafting of hydrophilic and hydrophobic polyester side chains, the macromolecules hydrophilicity or lipophilicity and self-assembly in water solutions can be well controlled.

The graft-copolymer composition affords the design of systems stimuli-responsive properties, including solubility, viscosity, and supramolecular association [16,27,28,29,30,31]. Given their brush-like architecture, graft copolymers display distinct stimuli-responsive behaviors. For example, for bottlebrush polymers with poly(*N*-isopropylacrylamide) (PNIPAAM) side chains reveal significant effect of the end group structure and side chain length on the behavior of aqueous solutions at varying temperature [31]. Hydrophobic end groups lead to poor solubility in water and lower the phase separation temperature *T_ph.tr._*, whereas the lower critical solution temperature (LCST) of samples with thiol-terminated side-chains is shown to be higher than for PNIPAAM homopolymers. Side chain elongation results in a decrease of the LCST. At *T* < *T_ph.tr._*, molecular dimensions of PNIPAAM bottlebrushes decrease with heating, followed by the formation of lyotropic liquid crystal phase at *T* > *T_ph.tr._* polymers. The LCST of poly(ethylene oxide)-graft-poly(*N*,*N*-dimethylaminoethyl methacrylate) (PEO-g-PDMAEMA) strongly decreases with increasing pH, yet remains lower than that of linear block copolymer counterparts [28]. By varying the pH and NaCl concentrations of PEO-g-PDMAEMA solutions, aggregates of various topologies can be obtained, e.g., worm-shaped cylindrical structures at low pH.

Due to their structural relationship to polypeptides, poly(2-alkyl-2-oxazoline)s (PAOx) are regarded as pseudopeptides whose properties strongly depend on the chemical structure of a substituent at the oxazoline moiety [32]. The tertiary amide groups in PAOx provide their stability in biological environments, which promotes their application in medicine and biotechnology [33,34,35,36,37]. Significant advancements are expected for PAOxs with complex architectures, in particular graft copolymers with PAOx side chains. Therefore, establishing and understanding comb-like PAOxs behaviors that distinguish them from linear analogues is an important task [38,39,40,41,42,43,44,45]. For example, graft copolymers with a methacrylate backbone and poly(2-ethyl-2-oxazoline) (PEtOx) side chains demonstrated coexistence of macromolecules aggregates at low temperatures [38]. Furthermore, the cloud point *T_cp_* was shown to decrease with increasing molar mass (MM). At *T > T_cp_*, contraction of the macromolecules was observed, but their conformation did not change. Jordan and co-workers prepared thermosensitive molecular brushes with PAOx side chains and showed that *T_cp_* decreased with increasing backbone length [39], while displaying non-linear dependence on the side chain length [11]. By using the polymerization-polycondensation approach, the same group of researchers synthesized stimulus-sensitive grafted copolymers with an aromatic polyester (APE) backbone and PEtOx side chains [46,47]. It was shown that the grafting density of the side chains *z* determines the self-organization mechanism of APE-g-PEtOx molecules, namely, compaction or aggregation, and causes a change in the phase separation temperature, which increases with *z*.

**Figure 1:**
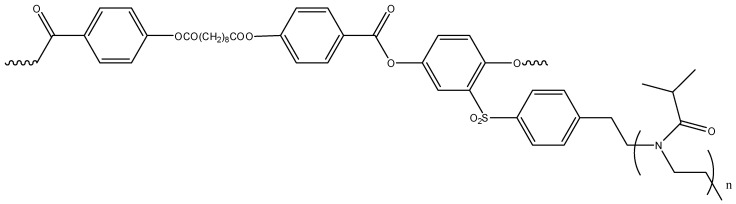
Structural formula of the aromatic polyester (APE)-g-poly-(2-isopropyl-2-oxazoline) (PiPrOx) graft copolymer.

It is of interest to find out how the replacement of side chains with more hydrophobic poly-(2-isopropyl-2-oxazoline) (PiPrOx) would affect the solution behavior of polymerization-polycondensation graft copolymers. Note that the thermal sensitivity of PEtOx and PiPrOx differs significantly. The dehydration of 2-ethyl-2-oxazoline units starts at 50 °C, and in the case of 2-isopropyl-2-oxazoline this temperature is about 20 °C [48]. Therefore, it is expected that grafting PiPrOx onto hydrophobic APE will lower the temperature of the onset of phase separation. The goal of this study is to synthesize a new APE-g-PiPrOx graft copolymer (Figure 1) and to analyze its molecular conformational self-organization behaviors in aqueous solutions upon heating. In addition, linear PiPrOx with a hydrophobic terminal group is synthesized as a reference polymer that models side chains to be compared with graft copolymers with the same side chains.

## 2. Materials and Methods

### 2.1. APE-g-PiPrOx Molecular Brush Synthesis

2-[4-(2-Br-ethyl)]phenylsulfonylhydroquinone (**1**) [49], 4,4′-sebacyloyldioxydibenzoic acid, and 4,4′-sebacyloyldioxydibenzoyldichloride (**2**) [50] were synthesized according to the known procedures. 2-isopropyl-2-oxazoline, diphenyloxide, and 1,1,2,2-tetrachloroethane (Aldrich, St. Louis, MO, USA) were dried over calcium hydride and distilled.

The NMR spectra were recorded on the Bruker AC 400 spectrometer (400 MHz) for solutions in CDCl_3_. Dialysis was performed using dialysis bags (CellaSep, Orange Scientific, Wallonia, Belgium) with the MWCO of 3500 g·mol^−1^.

The chromatographic analysis was performed on the Shimadzu LC-20AD chromatograph equipped with TSKgel G5000HHR column (5 μm, 7.8 mm × 300 mm, Tosoh Bioscience, Tokyo, Japan) and a refractometric detector. A solution of LiBr in DMF (0.1 mol/L) at 60 °C was used as the mobile phase. Calibration was performed relative to poly(ethylene glycol) standards (*M*_w_ = 6 × 10^2^–4 × 10^4^ g·mol^−1^).

Poly(2-[4-(2-Br-ethyl)phenylsulfonyl]-1,4-phenylene-4′,4″-sebacylyldioxydibenzoate) (**3**). A flask equipped with a stirrer and a gas-supplying tube was charged with 1 (4.23 g, 0.01 mol), 3c (4.79 g, 0.01 mol), and diphenyl oxide (30 mL). The obtained mixture was purged with dry argon and heated up to 200°C under a flow of gas. The reaction mixture was kept at 200 °C for 2 h. The polymer was precipitated with hexane, continuously extracted with hexane in Soxlet apparatus during 6 h and dried. Yield 6.5 g (92%). ^1^H NMR (CDCl_3_, ppm.): 1.39 (m, COCH_2_CH_2_CH_2_CH_2_) 1.86 (t, COCH_2_CH_2_), 2.68 (t, COCH_2_CH_2_), 3.22 (d, ArCH_2_CH_2_Br), 3.56 (d, ArCH_2_CH_2_Br), 7.11–8.43 (m, Ar–H).

Polymerization of 2-isopropyl-2-oxazoline on the polyester macroinitiator. The solution of the initiator and monomer in 1,1,2,2-tetrachloroethane (feed ratio monomer/functional groups of macroinitiator 30/1) was heated under argon during 72 h at 70 °C. The solvent was distilled off in the vacuum polymer was dissolved in ethanol, dialyzed against water during 48 h and freeze-dried.

### 2.2. Synthesis of Linear PiPrOx-m with a Hydrophobic Terminal Group

2-[4-(2-Br-ethyl)]phenylsulfonylhydroquinone dibenzoate (**5**). 3 g (21 mmol) of benzoyl chloride was added to the solution of 3.5 g (20 mmol) 2-[4-(2-Br-ethyl)]phenylsulfonylhydroquinone (**4**) in 20 mL of dry pyridine under vigorous stirring at 0 °C. The reaction mixture was kept at room temperature for 12 h and poured into a 10% hydrochloric acid solution. 2-[4-(2-Br-ethyl)]phenylsulfonylhydroquinone dibenzoate was filtered off and recrystallized from toluene. Yield 5.3 g (74%). ^1^H NMR (CDCl_3_, ppm.): 3.22 (d, ArCH_2_CH_2_Br), 3.56 (d, ArCH_2_CH_2_Br), 7.31–8.17 (m, Ar–H).

Polymerization of 2-isopropyl-2-oxazoline using **5** as initiator. The solution of 56 mg (0.1 mmol) of **5** and 3.4 g (30 mmol) of 2-isopropyl-2-oxazoline in 4 mL of acetonitrile was heated under argon during 72 h at 70 °C. The reaction mixture was diluted with ethanol, dialyzed against water during 48 h and freeze-dried. ^1^H NMR (CDCl_3_, ppm.): 1.27 (b.s CH_3_) 2.92, 2.73 (rotamers d CH(CH_3_)_2_) 3.47 (b.s CH_2_CH_2_N) 7.31–8.17 (m, Ar–H).

### 2.3. Study of Behavior of the Synthesized Polymers in Organic Solvents

Molecular weight and hydrodynamic properties of APE-g-PiPrOx and PiPrOx-m were characterized in dilute solutions in nitropropane at 21 °C. The weight average molecular weight *M*_w_ and hydrodynamic radii *R*_h-D_ were measured by static and dynamic light scattering using a Photocor Complex instrument (Photocor Instrument Inc., Moscow, Russia), equipped with a Photocor-PC2 correlator with 288 channels and a Photocor-PD detector for measuring transmitted light intensity. A Photocor-DL semiconductor laser with a wavelength of λ_0_ = 659.1 nm was used as a light source. Toluene was used as a calibration fluid with an absolute scattering intensity of *R*_v_ = 1.38 × 10^−5^ cm^−1^. Prior to light scattering measurements, the solutions were filtered into dust-free cells using Chromafil polyamide filters (Macherey-Nagel GmbH & Co. KG, Düren, Germany) with the pore size of 0.45 μm. The values of the refractive index increment dn/dc were measured on the RA-620 refractometer (KEM, Kyoto, Japan).

**Figure 2:**
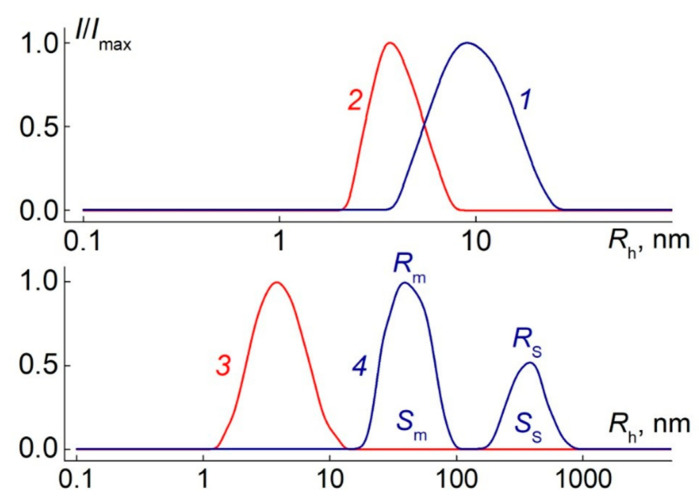
The dependences of relative intensity *I*/*I*_max_ of scattered light on the hydrodynamic radius *R*_h_ of scattering species for solutions of APE-g-PiPrOx at *c* = 0.0073 g·cm^−3^ (**1**) and PiPrOx-m at *c* = 0.0357 g·cm^−3^ (**2**) in nitropropane; for solutions of PiPrOx-m at *c* = 0.0020 g·cm^−3^ (**3**) and APE-g-PiPrOx at *c* = 0.00010 g·cm^−3^ (**4**) in water. *I*_max_ is the maximum value of light scattering intensity *I* for given solution concentration; explanations for *R*_m_, *R*_s_, *S*_m_, and *S*_s_ see below in Section 3.3.

At all concentrations, the scattered light intensity distribution was unimodal (Figure 2). The hydrodynamic radii *R*_h-D_(*c*) determined at concentration *c* in nitropropane did not depend on *c* (Figure S1), and the average value of *R*_h-D_(*c*) was taken as the hydrodynamic radius of the macromolecules *R*_h-D_.

For both APE-g-PiPrOx and PiPrOx-m samples, scattered light asymmetry was absent, and the MM was determined by the Debye method using the following equation

(1) $$\frac{cH}{I}=\;\frac{1}{M_{w}}+2A_{2}c$$

where *I*_90_ is the intensity of the light scattering at the 90° angle, *A*_2_ is the second virial coefficient, and *H* is the optical constant. The value of *H* was calculated as

(2) $$H=\frac{4\pi^{2}n_{0}^{2}{\left(dn/dc\right)}^{2}}{N_{A}\lambda_{0}^{4}}$$

where *n*_0_ = 1.3943 is the solvent refractive index and *N*_A_ is the Avogadro’s number. Figure S2 shows the dependence of the inverse light scattering intensity on solution concentration *c*. A positive slope the dependences of *cH/I*_90_ on *c* indicates that nitropropane is a thermodynamically good solvent for APE-g-PiPrOx and PiPrOx-m; the values of the second virial coefficient being *A*_2_ = 4.4 × 10^−4^ and 5.4 × 10^−4^ cm^3^·mol·g^−1^ for the first and the second, respectively.

The intrinsic viscosity was measured in nitropropane with the Ostwald-type Cannon-Manning capillary viscometer (Cannon Instrument Company Inc., State College, PA, USA) at 21 °C. The solvent efflux time was 48.5 s. The concentration range was (0.0026–0.0096) g/cm^3^ for PiPrOx-m and 0.020–0.039 for APE-g-PiPrOx. Ratios of solution to solvent efflux times were always above 1.13.

### 2.4. Study of Behavior of APE-g-PiPrOx and PiPrOx-m in Aqueous Solutions upon Heating

The self-organization processes of APE-g-PiPrOx and PiPrOx-m in aqueous solutions were studied by light scattering and turbidimetry using the Photocor Complex setup described above. The solution temperature T was changed in increments ranging from 5 to 1 K at low temperatures and near the clouding point, respectively. The temperature was controlled with an accuracy of 0.1 °C.

Thermal variations of scattered and transmitted light intensities (*I* and I*) were used to obtain phase separation temperatures. In addition, hydrodynamic radii *R*_h_ of scattering particles and their contribution *S*_i_ to the total scattering intensity were measured as a function of temperature. The value of *S*_i_ was estimated as the area under the corresponding distribution peak *I*(*R*_h_). Particle sizes and their contributions were measured after reaching the equilibrium state of the solutions. Under these conditions, the angular dependences of the *I*, *R*_h_ and *S*_i_ values in the range of angles of light scattering from 45° to 135° were investigated in order to prove the diffusive nature of the modes, as well as to obtain extrapolated values of *R*_h_ and *S*_i_, which were used in further discussion.

## 3. Results and Discussion

### 3.1. Synthesis

**Figure 3:**
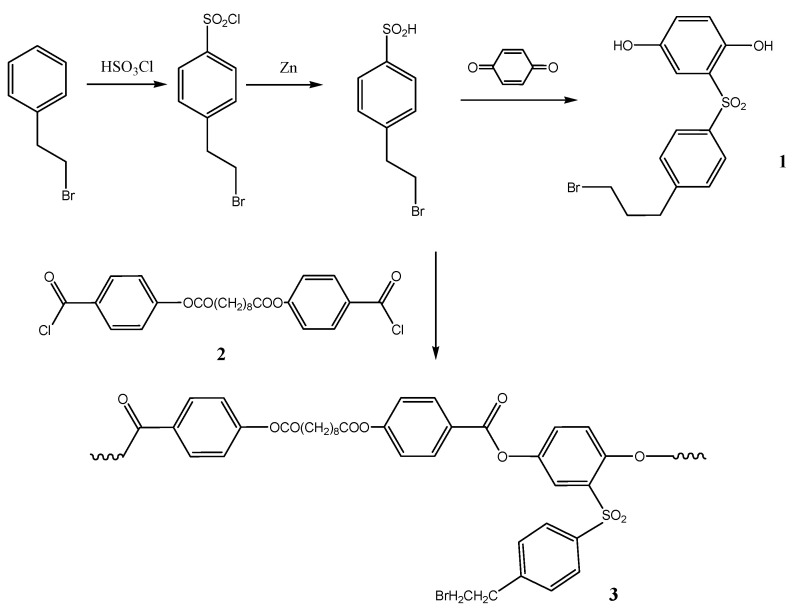
Synthesis route of APE (**3**) ie chlorosulfonation of 2-bromoethylbenzene, reduction of arenesulfonylchloride to sulfinic acid, Michael addition the latter to quinone leading to 2,5-dihydroxy-4′-(2-bromoethyl)-diphenylsulfone (**1**) and its polycondensation with 4,4′-sebacyloyldioxydibenzoyl dichloride (**2**).

For the synthesis of polyester-graft-polyoxazoline brushes [46], we first synthesized multicenter macroinitiator based on 2-[4-(2-bromoethyl)]phenylsulfonylhydroquinone and 4,4′-sebacyloyldioxydibenzoic acid (Figure 3).

**Figure 4:**
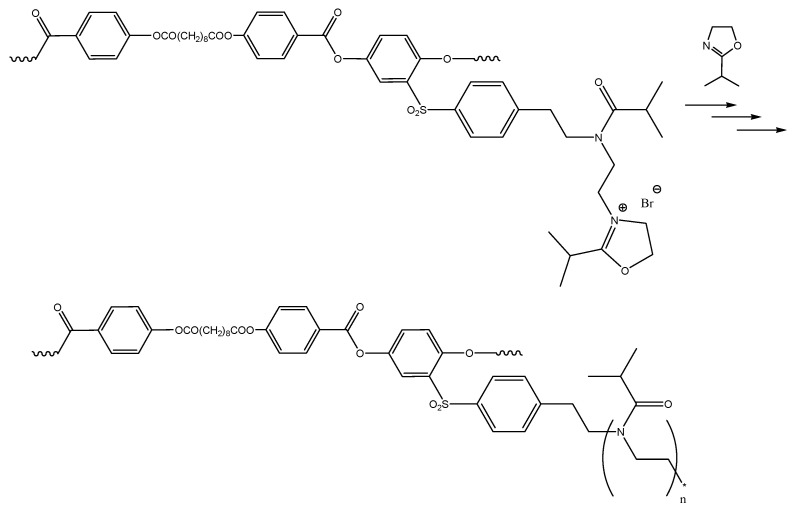
Synthesis of the poly(2-[4-(2-ethylene)phenylsulfonyl]-1,4-phenylene-4′,4″-sebacylyldioxydibenzoate-*graft*-poly-*N*-isoburyroythyleneimine.

Compound **1** was synthesized using the Spinner method, which involves chlorosulfonation of 2-phenethyl bromide, reduction of the corresponding sulfochloride to sulfinic acid, and addition of the latter to 1,4-benzoquinone. For the macroinitiator synthesis we used high-temperature acceptorless polycondensation [51], which yielded regular copolyesters with polydispersity indexes at about 2.1–2.3. The targeted graft copolymer APE-g-PiPrOx was synthesized under conditions that are conventionally used for polyoxazolines (Figure 4).

**Figure 5:**
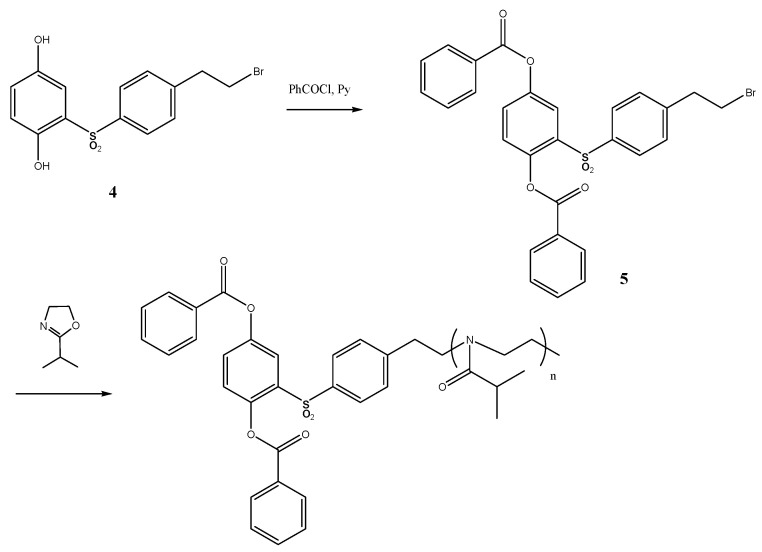
Synthesis of the model poly(2-isopropyl-2-oxazoline).

A scheme of the linear model polyester PiPrOx-m synthesis is shown in Figure 5. Keeping in mind the end group effect on thermoresponsiveness, compound **5**, whose structure was the closest to that of the macroinitiator, was synthesized as an initiator for the model polymer synthesis. For this purpose, the hydroxyl groups **1** (given in Figure 3) were blocked by benzoyl groups under Einhorn reaction conditions allowing for high yield of the targeted compound.

Comparison of ^1^H NMR spectra of the macroinitiator and graft copolymer shows that the copolymer spectrum exhibits both signals from aromatic protons in the backbone and from poly(2-isopropyl-2-oxazoline) side chains (Figure S3). Given the unimodal molecular-mass distribution of the obtained polymer (Figure S4), this suggested that the synthesized sample was a graft copolymer.

### 3.2. Structural and Molecular Characteristics of Grafted Copolymer APE-g-PiPrOx and Linear PiPrOx-m

Molecular and hydrodynamic characteristics obtained for APE-g-PiPrOx and PiPrOx-m are given in Table 1. As is known [52], the density of side chain grafting affects a number of physical characteristics of comb-like polymers, in particular, their behavior in solution. For determining side chains molar mass as well as their grafting density, polymer samples were subjected to alkali hydrolysis under conditions providing the quantitative scission of the polyester chain: 1 M KOH solution in ethyl 2-ethoxyethanol at 120 °C for 10 min [53]. The density of side chain grafting was found from the molar mass ratio of the backbone and side chains. With regular distribution of the initiating groups throughout the macroinitiator chain, the grafting density was determined by polymerization initiation efficiency. This parameter reflects the fraction of the macroinitiator initiating groups having reacted from the total content of the initiating groups, which was calculated as the ratio of the theoretical molar mass of side chains to the experimental value [54]. In the model experiment, under similar conditions, poly-2-ethyl-2-oxazoline was stable for at least 1 h. It was found that oligo-2-isopropyl-2-oxazoline side chains had *M*_s_ = (4300 ± 600) g·mol^−1^ and polydispersity *M*_w_/*M*_n_ = 1.45 ± 0.19.

Grafting density *z* was calculated as

*z* = *N*_1_/(*N*_1_ + *N*_2_) = (*M*_cop_ − *M*_b_)/*M*_s_ (*DP*_APE_) (3)

where *N*_1_ + *N*_2_ = *DP*_APE_ is the polymerization degree of APE macroinitiator, *N*_1_ and *N*_2_ are respectively the numbers of monomeric units of APE which contain or not contain PiPrOx side chain, *M*_cop_ is molar mass of APE-g-PiPrOx copolymer (Figure 1), and *M*_b_ = 19,000 g·mol^−1^ [55] is the backbone molar mass. According to calculations, *z* = 0.49, i.e., one side chain corresponded to two monomeric units of the copolymer. However, the APE monomers were very long, and in terms of the number of valence bonds, the grafting density was z* = 1/30 = 0.03, which suggested that APE-g-PiPrOx graft copolymer should be considered as a polymer comb [56], or as they are also called, loosely grafted molecular brushes [57].

**Figure 6:**
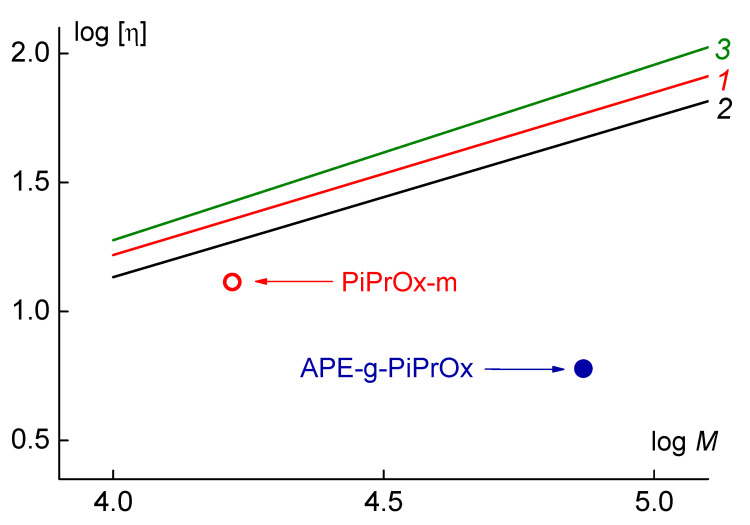
Intrinsic viscosity [η] vs. molar mass *M* for the studied polymers in nitropropane (data points), and Mark–Kuhn–Houwink–Sakurada (MKHS dependences for linear poly(2-ethyl-2-oxazoline) (PEtOx) (lines 1 [58] and 2 [59]) and poly(2-methyl-2-oxazoline) (PMeOx) (line 3 [58]).

The intrinsic viscosity [η] and hydrodynamic radius *R*_h_ of the APE-g-PiPrOx graft copolymer and the linear PiPrOx were analyzed using the Mark–Kuhn–Houwink–Sakurada (MKHS) relationships obtained for some poly(2-alkyl-2-oxazolines) in [58,59] (see Figure 6). As is known, the intrinsic viscosity [η] is related to the molecular weight by the MKHS equation [η] = K_x_M^a^, where the parameter K_x_ and the exponent a are constant values for a given polymer-solvent system. The value of [η] depends on both the size (MM) and the shape (conformation) of the macromolecule. The linear PiPrOx-m demonstrate a [η] value similar to that of poly(2-ethyl- and poly(2-methyl-2-oxazolines) given in literature [58,59], which is consistent with the similar chain stiffness. Therefore, it can be assumed that the conformational properties of PiPrOx-m with a molecular weight close to PAOx shown in the graph were similar. In contrast, the significantly lower [η] of the APE-g-PiPrOx graft copolymer suggests that their macromolecules were more compact than linear poly(2-alkyl-2-oxazoline)s.

These findings are consistent with our previous study of PI-g-PMMA and PI-g-PS graft copolymers with polyimide (PI) backbone and polymethylmethacrylate (PMMA) and polystyrene (PS) side chains in selective solvents [25]. The intrinsic viscosity of PI-g-PMMA and PI-g-PS did not depend on MM in the range from 270,000 to 2,000,000 g·mol^−1^, and the [η] value for grafted copolymer solutions was 4–8 times lower than the intrinsic viscosity of linear PMMA (*M*_w_ = 490,000 g·mol^−1^) and PS (*M*_w_ = 370,000 g·mol^−1^). This behavior is explained by the fact that in selective solvents where the main chain does not dissolve, the macromolecules of loose cylindrical brushes resemble multiarm star-shaped molecules [24]. The core of the star is a collapsed main chain, and the arms are more or less folded side chains, which should be considered as a corona ensuring the solubility of the copolymer.

**Figure 7:**
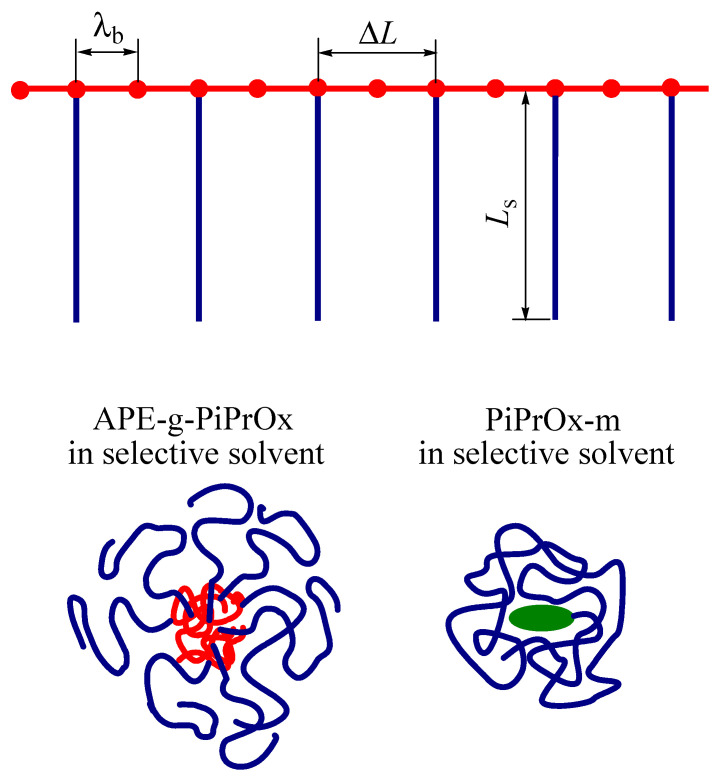
Scheme of the APE-g-PiPrOx molecules and conformation of molecules of APE-g-PiPrOx and linear PiPrOx-m in a selective solvent.

As shown by computer simulation [24], compact conformation requires (i) sufficiently high flexibility of the main chain and (ii) its access to the solvent. Both of these conditions were fulfilled for the studied APE-g-PiPrOx sample. The Kuhn segment length of APE did not exceed 2 nm, that is, the main chain was a typical flexible-chain polymer. On the other hand, the average distance Δ*L* between the grafted chains along the APE chain was not very different from the length *L*_s_ of the latter (Figure 7). Indeed,

ΔΔ*L* = *L*_b_/(*N*_1_ + *N*_2_)/*z* = λ_b_/*z* (4)

where *L*_b_ is the contour length of the main chain and λ_b_ is the length of the APE_8_ monomer unit. Assuming that the length of all valence bonds of the main chain was close to 0.14 nm, and the valence angles were tetrahedral, we obtained λ_b_ ≈ 3.5 nm and Δ*L* ≈ 7.7 nm. The average length of the PiPrOx side chains was *L*_s_ = λ_s_*N*_s_ = λ_s_*M*_PiPrOx_/*M*_s0_ ≈ ≈ 13.8 nm, where *M*_PiPrOx_ and *N*_s_ ≈ 37 are the MM and the polymerization degree of the grafted PiPrOx, *M*_s0_ = 112 g·mol^−1^ and λ_s_ = 0.378 nm are the molar mass and the length of PiPrOx monomer unit, respectively. Therefore, Δ*L* and *L*_s_ differed less than two times, and the length of the side chains was not sufficient to reliably shield the main chain from the solvent. This led to the collapse of the main chain, and the shape of the grafted copolymer molecule resembled a star-shaped molecule (Figure 7). Note that a similar conformation of comb-shaped macromolecules with a relatively short main chain was predicted by Tsvetkov over 30 years ago [60].

The behavior of APE-g-PiPrOx in nitropropane corresponded to that of a star-shaped molecule with a relatively massive core and the number of arms *f*_a_ corresponding to the number of PiPrOx grafted chains, namely, *f*_a_ = *z*(*N*_1_ + *N*_2_) ≈ 13. The length of arms *L*_s_ did not significantly differ from the hydrodynamic radius *R*_h-D_ of the APE-g-PiPrOx macromolecules. The ratio *L*_s_/*R*_h-D_ is *L*_s_/*R*_h-D_ = 1.5, which suggested that the grafted chains had the conformation of a slightly bent rod. This conclusion is in qualitative agreement with the predictions of Daoud and Cotton [61] theory describing the structure of multiarm polymer stars.

As for the model PiPrOx-m, its intrinsic viscosity and hydrodynamic radius were also lower than [η] and *R*_h_ for linear PAOx (Figure 6). Therefore, its molecules had a more compact structure compared to that of the PAOx molecules of the same MM. This is because the massive hydrophobic end group was insoluble, and the linear PiPrOx chain shielded it from the solvent, providing an increase in intramolecular density and a decrease in the size of PiPrOx-m molecules (Figure 7). The same was true for solutions of diphilic linear dendritic block copolymers [62,63,64,65,66]. The analysis of the results obtained for them made it possible to propose a solubility model of such hybrid systems [65]. In particular, it was shown that their behavior in solutions was significantly affected by both the thermodynamic quality of the solvent with respect to the blocks and the size ratios of the soluble linear chain and the insoluble branched component. The decrease in the length of the linear block at fixed sizes of the second block resulted in the appearance of supramolecular micelle-like structures in solutions, where insoluble components of different macromolecules form the core, and the linear chains wrapping around the latter ensure the solubility of the aggregates. In the case of the studied PiPrOx-m, the size of the terminal group is approximately 1.5–2.0 nm, the length of the linear PiPrOx chain is close to 54 nm, that is, it can “wrap” around the hydrophobic fragment almost 10 times. In contrast, the length of PiPrOx in the isolated side chain is insufficient to screen the end group, and large aggregates are formed in their solutions in nitropropane and water. To conclude the conformational analysis, we note that the size of poly-2-ethyl-oxazoline molecules in tetrahydrofuran [67] is smaller than in other solvents [58,59], and their shape is closer to spherical in the authors’ opinion. This is probably how the effect of the thermodynamic quality of the solvent on the solubility of PAOx is manifested.

### 3.3. Behavior of Grafted APE-g-PiPrOx Copolymer and Linear PiPrOx-m in Aqueous Solutions

In PiPrOx-m aqueous solutions at low temperatures, a single mode was observed by dynamic light scattering (Figure 2). The hydrodynamic radius *R*_h_ of particles in solution did not depend on concentration (Figure S5), while the average value of *R*_h_ = (3.9 ± 0.7) nm matched the hydrodynamic sizes of PiPrOx-m molecules in nitropropane. Therefore, at low temperatures, aqueous PiPrOx-m solutions were molecularly dispersed. This conclusion is also supported by good agreement between the PiPrOx-m molar mass *M*_w_ = (17,200 ± 900) g·mol^−1^, determined in both water at 21 °C and nitropropane (Figure S2). Thus, the presence of a massive hydrophobic group at one end of the model PiPrOx-m chain does not lead to formation of core-shell aggregates, including star-like and flower-like ones, which are often observed in solutions of linear amphiphilic PAOx [68,69,70,71,72,73]. Similar to nitropropane, hydrophobic fragments were completely shielded by long hydrophilic PiPrOx chains consisting of more than 140 monomer units.

In low temperature APE-g-PiPrOx solutions, two types of scattering objects with hydrodynamic radii *R*_m_ (Figure 2, line 4) and *R*_s_ (Figure 2, line 5) were found that significantly exceeded the size of the molecules of the grafted copolymer *R*_h-D_. This suggests particles aggregations due to attraction of hydrophobic APE main chains, as well as dehydrated PiPrOx units. The radii *R*_m_ and *R*_s_ decreased with dilution (Figure S5), which could reflect both the concentration dependence of the diffusion coefficient and the gradual destruction of aggregates with decreasing solution concentration.

It should be noted that at all concentrations the weight fraction of large aggregates with radius *R*_s_ was small. Indeed, the contribution of the *i*–th set of particles to the total light scattering intensity *I* is described by the relation

*I*_i_ ~ *c*_i_*R*_i_*^x^* (5)

where *c*_i_ and *R*_i_ are the weight concentration and radius of *i*-th particles, respectively [74]. The exponent *x* is determined by the shape of the particles: for hard rods *x* = 1, for coils *x* = 2 and for hard spheres *x* = 3. Using relation (4), we can roughly estimate the maximum value of the ratio *c*_s_/*c*_m_ of the weight concentrations of aggregates of large *c*_s_ and lower *c*_m_ sizes:

*c*_s_/*c*_m_ = *I*_s_*R*_m_*^x^*/*I*_m_*R*_s_*^x^* (6)

where *I*_s_ and *I*_m_ are contributions to the total light scattering of aggregates with radii *R*_s_ and *R*_m_, respectively. For the studied APE-g-PiPrOx solutions at low temperatures, the ratio of the aggregates contribution *I*_s_/*I*_m_ = *S*_s_/*S*_m_ to the total light scattering *I* ranges from 0.1 to 0.3. Here *S*_s_ and *S*_m_ are the areas under the peaks corresponding to aggregates with radii *R*_s_ and *R*_m_ on dependences of relative intensity *I*/*I*_max_ of scattered light on the hydrodynamic radius (Figure 2). Therefore, the maximum value of *S*_s_/*S*_m_ = 0.3, while the minimum value of the ratio of aggregate radii *R*_s_/*R*_m_ = 7. Thus, assuming that the conformation of the aggregates is close to coil-like (*x* = 2), we obtain the maximum value *c*_s_/*c*_m_ ≈ 0.007. Therefore, more than 99% of the polymer molecules formed aggregates with the radius *R*_m_, while large aggregates contained a maximum of 0.6% of the total number of molecules.

Earlier, two types of particles were also found in solutions of the grafted APE-g-PEtOx copolymer with the polyester main chain and the side chains of poly(2-ethyl-2-oxazoline) at room temperature [46,47]. However, in the case of APE-g-PEtOx, these particles were grafted polymer molecules and aggregates similar in characteristics to smaller aggregates in APE-g-PiPrOx solutions. Considering that the studied APE-g-PiPrOx and APE-g-PEtOx samples had similar grafting densities z and side chain lengths *L*_s_, we concluded that the difference in the behavior of the compared polymers was due to the different hydrophilicity of PiPrOx and PEtOx. More hydrophobic PiPrOx chains contributed to more intense aggregation.

**Figure 8:**
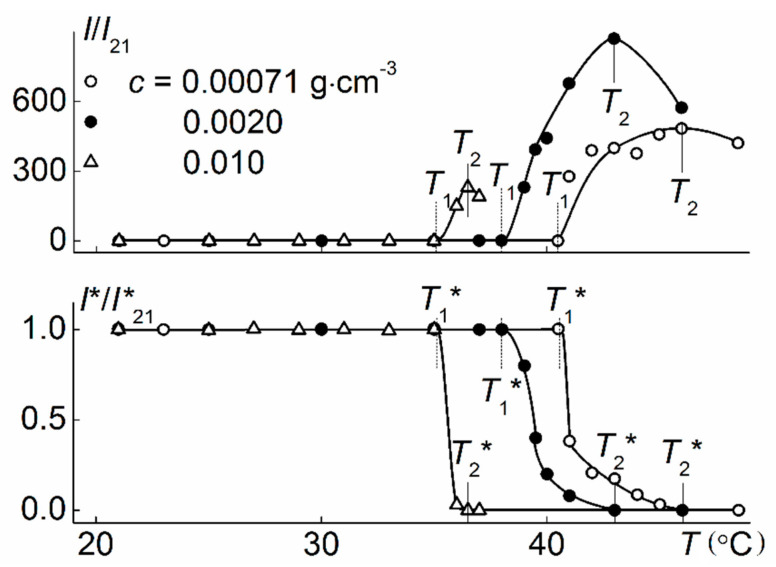
The temperature dependences of relative light scattering intensity *I*/*I*_21_ and relative transmittance I*/I*_21_ for aqueous solutions of PiPrOx-m. *I*_21_ and I*_21_ are intensities of light scattering and optical transmittance at *T* = 21 °C, respectively.

**Figure 9:**
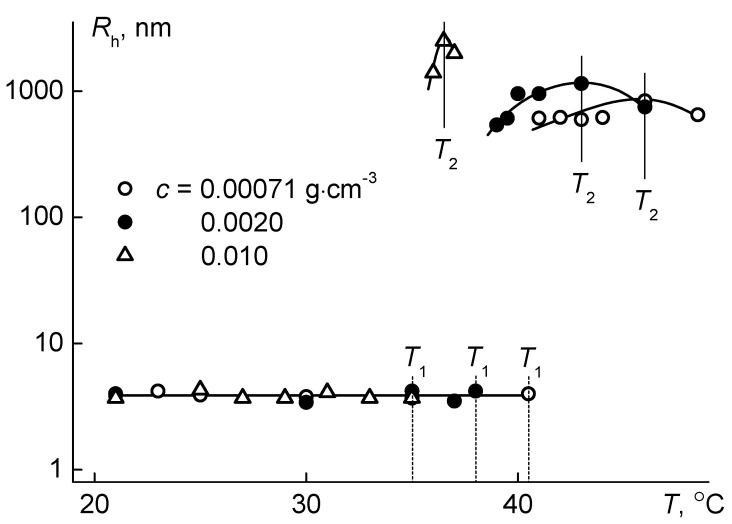
Dependences of hydrodynamic radii *R*_h_ of scattering species on temperature *T* for aqueous solutions of PiPrOx-m.

When heating PiPrOx-m aqueous solutions, phase separation was observed, which manifested itself in the sharp increase in the light scattering intensity *I* and the corresponding decrease in the optical transmittance I* (Figure 8). The profiles of the dependences *I*/*I*_21_(*T*) and I*/I*_21_(*T*) obtained for PiPrOx-m solutions of various concentrations were similar to those for thermosensitive polymers characterized by LCST behavior [35,71,75,76,77,78,79]. The temperatures of the phase separation onset, *T*_1_ and *T*_1_*, and termination, *T*_2_ and *T*_2_*, obtained from both dependences, coincided pairwise. The change in *I* and I* in the interval from *T*_1_ to *T*_2_ was due to the aggregation of macromolecules. Large micron aggregates appeared in solutions as a result of the formation of hydrophobic intermolecular contacts due to dehydration of PiPrOx-m units upon heating. The size of aggregates increased with increasing *T* in the interval of phase separation and reached the maximum values near *T*_2_ (Figure 9). Above *T*_2_, the size of the aggregates decreased, which may reflect the compaction of macromolecules, but we should remember that under these conditions the solution was cloudy and light scattering was not classical.

**Figure 10:**
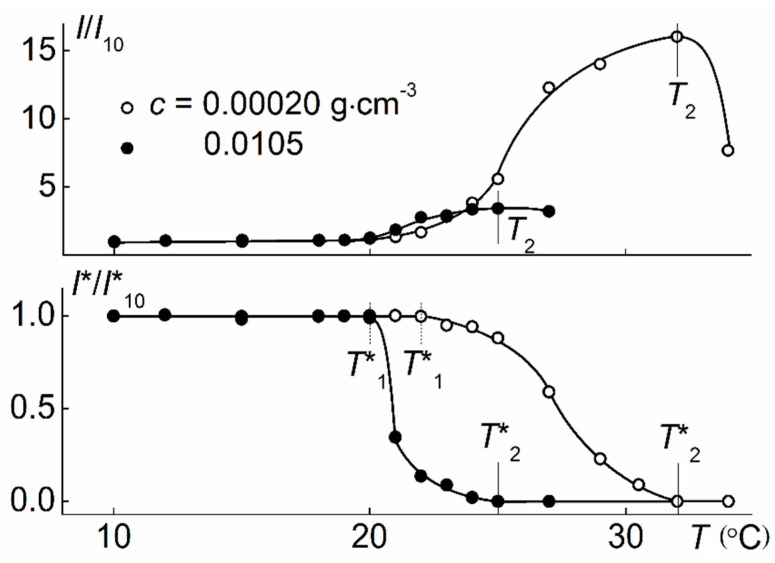
The temperature dependences of relative light scattering intensity *I*/*I*_10_ and relative transmittance I*/I*_10_ for aqueous solutions of APE-g-PiPrOx. *I*_10_ and I*_10_ are intensities of light scattering and optical transmittance at *T* = 10 °C, respectively.

Figure 10 shows the dependences of light scattering intensity *I* for aqueous solutions of APE-g-PiPrOx. The value of *I* monotonically increased with temperature at an increasing rate d*I*/d*T*, which does not allow determining the temperature of the onset of phase separation *T*_1_ using light scattering data. The intensity *I* reached its maximum value at temperature *T*_2_, which for all studied solutions was similar to T*_2_, corresponding to the termination of phase separation according to the turbidity data (Figure 10). The temperatures of the phase separation onset *T*_1_ were determined by the beginning of the I* decline.

**Figure 11:**
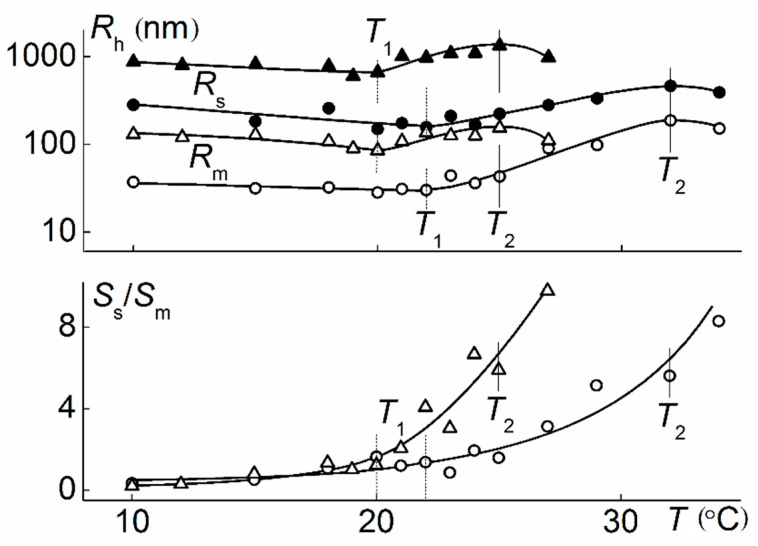
The temperature dependences of hydrodynamic radii *R*_h_ and *S*_s_/*S*_m_ for middle (open signs) and slow (filled signs) scattering species at *c* = 0.00020 g·cm^−3^ (circles) and 0.0105 g·cm^−3^ (triangles) in APE-g-PiPrOx aqueous solutions.

As can be seen in 11, at *T < T*_1_, the hydrodynamic radii of the aggregates *R*_m_ and *R*_s_ slowly decreased upon heating, i.e., aggregates compacted in this temperature range due to dehydration of PiPrOx chains and the formation of hydrogen bonds, both on the intramolecular and intermolecular level. Note that the data obtained did not allow us to make a conclusion about which aggregates, large or small, changed their size more significantly. For example, at the concentration of *c* = 0.00020 g·cm^−3^, the radius *R*_s_ decreased noticeably faster, and at *c* = 0.0105 g·cm^−3^, *R*_m_ changed slightly more significantly (Figure 11). Compaction was accompanied by aggregation, as indicated by the increase in the ratio *S*_s_/*S*_m_ of the contributions of smaller *S*_m_ and large *S*_s_ aggregates to integral light scattering (Figure 11). As well as compaction, the formation of new aggregates was due to the occurrence of hydrogen bonds between the dehydrated PiPrOx units. Within the framework of the model of coil-shaped scattering objects, for a solution with *c* = 0.00020 g·cm^−3^, the ratio of the weight concentrations of aggregates *c*_s_/*c*_m_ varied from 0.006 at *T* = 10 °C to 0.4 at *T*_1_ and up to 1.0 at *T*_2_. Obviously, this estimation is approximate, since compaction may be accompanied by both shape and density variation of aggregates. Yet it correctly reflected the tendency towards phase separation of APE-g-PiPrOx solutions upon heating.

**Figure 12:**
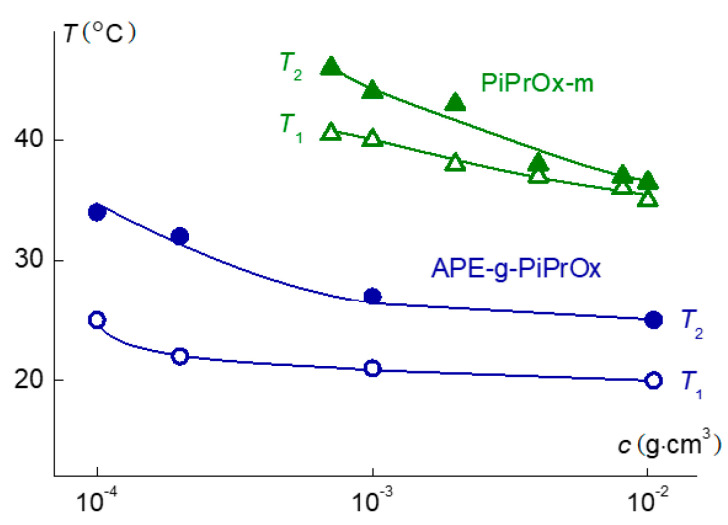
Concentration dependences of temperatures *T*_1_ and *T*_2_ for solutions of grafted copolymer APE-g-PiPrOx and model linear PiPrOx-m.

For both polymers investigated in this paper, the *T*_1_ and *T*_2_ temperatures as well as the phase separation range increase with dilution (Figure 12). For the linear model PiPrOx-m, a strong concentration dependence of phase separation temperatures is retained in the entire studied concentration range. The minimum value of *T*_1_ = 35 °C is close to LCST of solutions of linear PiPrOx [34,35,80,81]. For example, Uyama and Kobayashi [81] showed that for a PiPrOx sample with molar mass *M* = 16,700 g·mol^−1^, the phase transition temperature is ≈36 °C.

In the case of APE-g-PiPrOx gradt copolymer, the *T*_1_ and *T*_2_ dependences leveled off at high concentrations, suggesting that the LCST of the studied molecular brush was close to 20 °C, which was noticeably lower than typical LCST values for linear PiPrOx, including the studied PiPrOx-m. In addition to the difference in architecture between APE-g-PiPrOx and PiPrOx-m molecules, the observed LCST variation may be caused stronger hydrophobicity of the molecular brush and almost five-fold difference in the MM of the compared samples.

### 3.4. Kinetics of Thermal Response of APE8-g-PiPrOx and PiPrOx-m Solutions

All the results discussed above relate to the “equilibrium” state of solutions, that is, to a state where the characteristics of the solution do not change over time. There are many reports that after exposure to a stimulus, the parameters of stimuli-responsive systems gradually change in time [70,72,82,83,84,85], and for polymers with complex architecture, this process may last tens of hours [86].

It is convenient to use the time *t*_eq_, during which the light scattering intensity *I* and the optical transmission I* reach constant time values, as a characteristic of the duration of the “equilibrium” state of the system establishing (Figure S6). The time *t*_eq_ depends on the temperature, reaching a maximum value of $t_{\mathrm{eq}}^{\max}$ near the temperature *T*_1_ of the onset of phase separation (Figure S7). For both polymers, at *T* > *T*_1_, the value of *t*_eq_ rapidly decreased with heating. As mentioned above, for a molecular brush, the light scattering intensity I also depended on temperature at *T* < *T*_1_; therefore, for this polymer, in the given temperature range, the values of *t*_eq_ could be determined using the dependences of *I* on *t*. At low temperatures, *t*_eq_ rapidly increased with increasing *T*. Similar temperature dependences of the settling time *t*_eq_ were previously observed for polymers of various architectures, and are probably associated with intense aggregation at the beginning of phase separation [47,86].

**Figure 13:**
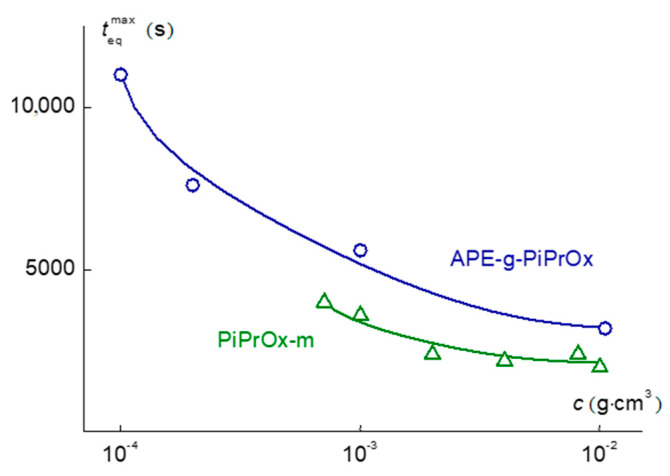
Concentration dependences of $t_{\mathrm{eq}}^{\max}\;$ for APE-g-PiPrOx and PiPrOx-m solutions.

It is unusual that for both polymers studied, $t_{\mathrm{eq}}^{\max}$ is highly concentration dependent, increasing with dilution (Figure 13). It can be assumed that this was due to the large sizes of aggregates at low concentrations. However, this issue requires additional research. The maximum time to establish an “equilibrium” state for APE-g-PiPrOx solutions was 11,000 s, which is comparable with $t_{\mathrm{eq}}^{\max}$ values for APE-g-PEtOx with a close MM [46,47] and for polymer brushes with polyimide backbone and poly (*N*,*N*-dimethylaminoethyl methacrylate) side chains [29], but noticeably less than $t_{\mathrm{eq}}^{\max}$ for star-shaped PAOx [86]. For model PiPrOx-m, the $t_{\mathrm{eq}}^{\max}$ times were noticeably lower, ranging from 2000 to 4000 s. The acceleration of self-organization processes for linear polymers compared to polymers of complex architecture was typical. However, in addition to this, an almost five-fold difference in the MM of the PiPrOx-m and APE-g-PiPrOx samples should be taken into account. Indeed, the molecular mass dependence of $t_{\mathrm{eq}}^{\max}$ can be quite strong. For example, for high molecular weight samples of statistical copolymers of *N*-isopropylacrylamide with ionogenic comonomers, the $t_{\mathrm{eq}}^{\max}$ values reached 9000 s [87].

## 4. Conclusions

New thermosensitive grafted copolymer was synthesized successfully. The backbone was polycondensation polymer, namely, an aromatic polyester. Polymerization poly(2-isopropyl-2-oxazoline), i.e., the polymer belonging to a fundamentally different chemical class, was used as side chain. This structure of the components determines the diphilicity of the resulting polymer in most solvents. APE-g-PiPrOx should be considered as a loose molecular brush because of their rather low density of side chains grafting (*z* = 0.49) and the ratio of the length of the PiPrOx side chains to the distance between the points of their grafting is less than 2. For comparison the linear PiPrOx sample with a hydrophobic terminal group modeling the side chain and a fragment of backbone of the grafted copolymer was synthesized.

Analysis of molar mass and hydrodynamic characteristics of the obtained polymers in organic solvent made it possible to conclude that the structures of the APE-g-PiPrOx molecules are more compact as compared to that of the PAOx molecules of the same MM. The conformation of the grafted copolymer macromolecule resembles a star, where the collapsed APE backbone forms a relatively massive core, and PiPrOx side chains serve as arms being in a folded conformation. The massive terminal group of the linear PiPrOx-m molecule is also shielded from the solvent by a sufficiently long linear PiPrOx chain which is wrapping around it forming a shell. This provides an increase in intramolecular density and a decrease in the size of PiPrOx-m macromolecules.

The thermosensitivity of both APE-g-PiPrOx and PiPrOx-m was shown. The phase separation of the APE-g-PiPrOx is preceded by the compaction of dissolved particles, namely aggregates of two types, as a result of dehydration of PiPrOx chains and formation of intramolecular and intermolecular hydrogen bonds. Above the phase separation temperature, aggregates sizes increase rapidly. The linear modelling PiPrOx-m exhibits the behavior usual for thermosensitive linear polymers.

The phase separation temperatures of the both polymers decrease with concentration. The LCST of the APE-g-PiPrOx under consideration, at given molecular and architectural characteristics, is assumed to be not much different from 20 °C, that is noticeably lower than typical LCST values for linear PiPrOx, including the studied PiPrOx-m. The difference in the APE-g-PiPrOx and PiPrOx-m thermosensitivity characteristics upon heating is conditioned not only by the specific architecture and the great hydrophobicity of the molecular brush, but also the almost five-fold difference in the MM of the compared polymers.

## Figures and Tables

**Table 1:** Molar mass, polydispersity index (PDI), degree of polymerization (DP) and hydrodynamic characteristics of grafted copolymer APE-g-PiPrOx, linear PiPrOx-m, APE macroinitiator, and abstracted side chains.

Polymer	*M*_w_ (g·mol^−1^)	*PDI*	*DP*	*R*_h_ (nm)	[η], cm^3^·g^−1^
APE-g-PiPrOx	74,000	2.6		9.5 ± 0.7	6.0 ± 0.3
PiPrOx-m	16,600	1.4		3.4 ± 0.3	13.0 ± 0.3
APE [55]	19,000		27	-	-
side chain	4300	1.45	37	-	-

## Supplementary Materials

The following are available online at https://www.mdpi.com/2073-4360/12/11/2643/s1, Figure S1: The concentration dependences of hydrodynamic radius Rh-D(c) for solutions of APE-g-PiPrOx (1) and PiPrOx-m (2) in nitropropane, Figure S2. The concentration dependences of light scattering intensity *I* for solutions of APE-g-PiPrOx (1) and PiPrOx-m (2) in nitropropane and PiPrOx-m in water (3), Figure S3. NMR spectra of APE macroinitiator (1) and graft copolymer APE-graft-PiPrOx, Figure S4. GPC trace of APE-graft-PiPrOx, Figure S5. The concentration dependences of hydrodynamic radii *R*_m_ and *R*_s_ for aqueous solutions of APE-g-PiPrOx and *R*_h_ for aqueous solutions of PiPrOx-m at low temperatures, Figure S6. Dependences of light scattering intensity *I*/*I*_0_ (1, 2) and optical transmission *I**/*I**_0_ (3, 4) on time *t* for APE-g-PiPrOx solutions at *c* = 0.00010 g cm−3 (1, 3) and for PiPrOx-m solutions at *c* = 0.0040 g cm−3 (2, 4). I0 and *I**_0_ are the intensity of light scattering and optical transmission at *t* = 0, respectively, Figure S7. Temperature dependences of teq for APE-g-PiPrOx and PiPrOx-m.

## References

[1] Zhang M., Müller A.H.E. (2005). Cylindrical polymer brushes. J. Polym. Sci. Part A Polym. Chem..

[2] Sheiko S.S., Sumerlin B.S., Matyjaszewski M. (2008). Cylindrical molecular brushes: Synthesis, characterization, and properties. Prog. Polym. Sci..

[3] Nakamura Y. (2016). Stiffness parameter of brush-like polymers with rod-like side chains. J. Chem. Phys..

[4] Dutta S., Wade M.A., Walsh D.J., Guironnet D., Rogers S.A., Sing C.E. (2019). Dilute solution structure of bottlebrush polymers. Soft Matter.

[5] Birshtein T.M., Borisov O.V., Zhulina Y.B., Khokhlov A.R., Yurasova T.A. (1987). Conformations of comb-like macromolecules. Polym. Sci. Ser. A.

[6] Wintermantel M., Schmidt M., Tsukahara Y., Kajiwara K., Kohjiya S. (1994). Rodlike combs. Macromol. Rapid Commun..

[7] Zhang B., Gröhn F., Pedersen J.S., Fischer K., Schmidt M. (2006). Conformation of Cylindrical Brushes in Solution:  Effect of Side Chain Length. Macromolecules.

[8] Terao K., Hokajo T., Nakamura Y., Norisuye T. (1999). Solution Properties of polymacromonomers consisting of polystyrene. Viscosity behavior in cyclohexane and toluene. Macromolecules.

[9] Ishizu K., Tsubaki K., Mori A., Uchida S. (2003). Architecture of nanostructured polymers. Progr. Polym. Sci..

[10] Borisov O.V., Zhulina E.B. (2005). Amphiphilic graft copolymer in a selective solvent: Intramolecular structures and conformational transitions. Macromolecules.

[11] Hadjichristidis N., Pitsikalis M., Pispas S., Iatrou H. (2010). Polymers with complex architecture by living anionic polymerization. Chem. Rev..

[12] Lagos A., Reyes J. (1988). Grafting onto chitosan: Graft copolymerization of methyl methacrylate onto chitosan with Fenton’s reagent as a redox initiator. J. Polym. Sci..

[13] Wataoka I., Urakawa H., Kobayashi K., Akaike T., Schmidt M., Kajiwara K. (1999). Structural characterization of glycoconjugate polystyrene in aqueous solution. Macromolecules.

[14] Tripathy J., Mishra D.K., Yadav M., Behari K. (2010). Synthesis, characterization and applications of graft copolymer (chitosan-g-*N*,*N*-dimethylacrylamide). Carbohyd. Polym..

[15] Liu W., Liu Y., Hao X., Zeng G., Wang W., Liu R., Huang Y. (2012). Backbone-collapsed intra- and inter-molecular self-assembly of cellulose-based dense graft copolymer. Carbohydr. Polym..

[16] Zakharova N.V., Simonova M.A., Zelinskii S.N., Annenkov V.V., Filippov A.P. (2019). Synthesis, molecular characteristics, and stimulus-sensitivity of graft copolymer of chitosan and poly(*N*,*N*-diethylacrylamide). J. Mol. Liquids.

[17] Liang M., Jhuang Y.-J., Zhang C.-F., Tsai W.-J., Feng H.-C. (2009). Synthesis and characterization of poly(phenylene oxide) graft copolymers by atom transfer radical polymerizations. Eur. Polym. J..

[18] Yilmaz G., Toiserkani H., Demirkol D.O., Sakarya S., Timur S., Yagci Y., Torun L.J. (2011). Modification of polysulfones by click chemistry: Amphiphilic graft copolymers and their protein adsorption and cell adhesion properties. Polym. Sci. A.

[19] Fu G.D., Kang E.T., Neoh K.G., Lin C.C., Liaw D.J. (2005). Rigid Fluorinated Polyimides with Well-Defined Polystyrene/Poly(pentafluorostyrene) Side Chains from Atom Transfer Radical Polymerization. Macromolecules.

[20] Meleshko T.K., Ilgach D.M., Bogorad N.N., Kukarkina N.V., Vlasova E.N., Dobrodumov A.V., Malakhova I.I., Gorshkov N.I., Krasikov V.D., Yakimanskii A.V. (2010). Synthesis of multicentered polyimide initiators for the preparation of regular graft copolymers via controlled radical polymerization. Polym. Sci. Ser. B.

[21] Ilgach D.M., Meleshko T.K., Yakimansky A.V. (2015). Methods of controlled radical polymerization for the synthesis of polymer brushes. Polym. Sci. Ser. C.

[22] Meleshko T., Il’gach D., Bogorad N., Kukarkina N., Yakimansky A. (2014). Synthesis of graft copolyimides via controlled radical polymerization of methacrylates with a polyimide macroinitiators. Polym. Sci. Ser. B.

[23] Filippov A.P., Belyaeva E.V., Meleshko T.K., Yakimansky A.V. (2014). Solution behavior of polyimide-graft-polystyrene copolymers in selective solvents. J. Polym. Sci. Part B Polym. Phys..

[24] Filippov A.P., Belyaeva E.V., Krasova A.S., Simonova M.A., Meleshko T.K., Ilgach D.M., Bogorad N.N., Yakimansky A.V., Larin S.V., Darinskii A.A. (2014). Conformations of molecular brushes based on polyimide and poly(methyl methacrylate) in selective solvents: Experiment and computer simulation. Polym. Sci. Ser. A.

[25] Filippov A.P., Krasova A.S., Tarabukina E.B., Meleshko T.K., Yakimansky A.V., Sheiko S.S. (2018). Behavior of amphiphilic molecular brushes with polyimide main chain and side chains of polymethylmethacrylate and polystyrene in the vicinity of θ-point. Polym. Sci. Ser. C.

[26] Bilal M.H., Alaneed R., Steiner J., Mäder K., Pietzsch M., Kressler J. (2019). Chapter Three-Multiple grafting to enzymatically synthesized polyesters. Methods Enzymol..

[27] Luo Y.-L., Yuan J.-F., Liu X.-J., Xie H., Gao Q.-Y. (2010). Self-assembled polyion complex micelles based on PVP-b-PAMPS and PVP-b-PDMAEMA for drug delivery. J. Bioact. Comp. Polym..

[28] Sui K., Zhao X., Wu Z., Xia Y., Liang H., Li Y. (2012). Synthesis, rapid responsive thickening, and self-assembly of brush copolymer poly(ethylene oxide)-graft-poly(*N*,*N*-dimethylaminoethyl methacrylate) in aqueous solutions. Langmuir.

[29] Filippov A.P., Belyaeva E.V., Zakharova N.V., Sasina A.S., Ilgach D.M., Meleshko T.K., Yakimansky A.V. (2015). Double stimuli-responsive behavior of graft copolymer with polyimide backbone and poly(*N*,*N*-dimethylaminoethyl methacrylate) side chains. Colloid Polym. Sci..

[30] Wessels M.G., Jayaraman A. (2019). Molecular dynamics simulation study of linear, bottlebrush, and star-like amphiphilic block polymer assembly in solution. Soft Matter.

[31] Li X., ShamsiJazeyi H., Pesek S.L., Agrawal A., Hammouda B., Verduzco R. (2014). Thermoresponsive PNIPAAM bottlebrush polymers with tailored side-chain length and end-group structure. Soft Matter.

[32] De Laittre G. (2019). Telechelic poly(2-oxazoline)s. Eur. Polym. J..

[33] Fael H., Rafols C., Demirel A.L. (2018). Poly(2-Ethyl-2-Oxazoline) as an Alternative to Poly(Vinylpyrrolidone) in Solid Dispersions for Solubility and Dissolution Rate Enhancement of Drugs. J. Pharm. Sci..

[34] Hoogenboom R. (2009). Poly(2-oxazoline)s: A polymer class with numerous potential applications. Angew. Chem. Int. Ed..

[35] Hoogenboom R., Schlaad H. (2017). Thermoresponsive poly(2-oxazoline)s, polypeptoids, and polypeptides. Polym. Chem..

[36] de la Rosa V.R. (2014). Poly(2-oxazoline)s as materials for biomedical applications. J. Mater. Sci. Mater. Med..

[37] Rossegger E., Schenk V., Wiesbrock F. (2013). Design strategies for functionalized poly(2-oxazoline)s and derived materials. Polymers.

[38] Weber C., Rogers S., Vollrath A., Hoeppener S., Rudolph T., Fritz N., Hoogenboom R., Schubert U.S. (2013). Aqueous solution behavior of comb-shaped poly(2-ethyl-2-oxazoline). J. Polym. Sci. Part A Polym. Chem..

[39] Zhang N., Luxenhofer R., Jordan R. (2012). Thermoresponsive poly(2-oxazoline) molecular brushes by living ionic polymerization: Kinetic investigations of pendant chain grafting and cloud point modulation by backbone and side chain length variation. Macromol. Chem. Phys..

[40] Bühler J., Muth S., Fischer K., Schmidt M. (2013). Collapse of cylindrical brushes with 2-isopropyloxazoline side chains close to the phase boundary. Macromol. Rapid Commun..

[41] Weber C., Wagner M., Baykal D., Hoeppener S., Paulus R.M., Festag G., Altuntas E., Schacher F.H., Schubert U.S. (2013). Easy access to amphiphilic heterografted poly(2-oxazoline) comb copolymers. Macromolecules.

[42] Alvaradejo G.G., Nguyen H.V.-T., Harvey P., Gallagher N.M., Le D., Ottaviani M.F., Jasanoff A., Delaittre G., Johnson J.A. (2019). Polyoxazoline-Based Bottlebrush and Brush-Arm Star Polymers via ROMP: Syntheses and Applications as Organic Radical Contrast Agents. ACS Macro Lett..

[43] Nuyken O., Rueda-Sanchez J., Voit B. (1997). Synthesis of graft copolymers by ring-opening polymerization of 2-nonyl-and 2-phenyl-2-oxazoline initiated by macroinitiators containing benzylchloride functions. Polym. Bull..

[44] Rueda J., Zschoche S., Komber H., Schmaljohann D., Voit B. (2005). Synthesis and characterization of thermoresponsive graft copolymers of NIPAAm and 2-alkyl-2-oxazolines by the “grafting from” method. Macromolecules.

[45] Jerca V.V., Nicolescu F.A., Vasilescu D.S., Vuluga D.M. (2011). Synthesis of a new oxazoline macromonomer for dispersion polymerization. Polym. Bull..

[46] Kudryavtseva A.A., Kurlykin M.P., Tarabukina E.B., Tenkovtsev A.V., Filippov A.P. (2017). Behavior of thermosensitive graft-copolymers with aromatic polyester backbone and poly-2-ethyl-2-oxazoline side chains in aqueous solutions. Int. J. Polym. Anal. Char..

[47] Filippov A., Tarabukina E., Kudryavtseva A., Fatullaev E., Kurlykin M., Tenkovtsev A. (2019). Molecular brushes with poly-2-ethyl-2-oxazoline side chains and aromatic polyester backbone manifesting double stimuli responsiveness. Colloid Polym. Sci..

[48] Katsumoto Y., Tsuchiizu A., Qiu X.P., Winnik F.M. (2012). Dissecting the Mechanism of the Heat-Induced Phase Separation and Crystallization of Poly(2-isopropyl-2-oxazoline) in Water through Vibrational Spectroscopy and Molecular Orbital Calculations. Macromolecules.

[49] Spinner H., Yannopoulis J., Metamonski W. (1961). Oxidation-reduction polymers: I. Synthesis of monomers. Can. J. Chem..

[50] Bilibin A.Y., Tenkovtsev A.V., Skorokhodov S.S. (1985). Thermotropic polyesters, Synthesis of complex monomers for polycondensations. Makromol. Chem. Rapid Commun..

[51] Bilibin A.Y., Tenkovtsev A.V., Piraner O.N., Skorokhodov S.S. (1989). Investigation of the possibility of transesterification in the polycondensation of dihydroxyl compounds with acid dichlorides containing an ester bond. Makromol. Chem. Rapid Commun..

[52] Paturej J., Sheiko S.S., Panyukov S., Rubinshtein M. (2016). Molecular structure of bottlebrush polymers in melts. Sci. Adv..

[53] Allen B.J., Elsea G.M., Keller K.P., Kinder H.D. (1977). Quantitative hydrolysis-gas chromatographic methods for the determination of selected acids and glycols in polyesters. Anal. Chem..

[54] Sumerlin B.S., Neugebauer D., Matyjaszewski K. (2005). Initiation Efficiency in the Synthesis of Molecular Brushes by Grafting from via Atom Transfer Radical Polymerization. Macromolecules.

[55] Kurlykin M.P., Bursian A.E., Golub O.V., Filippov A.P., Tenkovtsev A.V. (2016). Multicenter polyester initiators for the synthesis of graft copolymers with oligo(2-ethyl-2-oxazoline) side chains. Polym. Sci. Ser. B.

[56] Liang H., Morgan B.J., Xie G., Martinez M., Zhulina E.B., Matyjaszewski K., Sheiko S.S., Dobrynin A.V. (2018). Universality of the entanglement plateau modulus of comb-like and bottlebrush polymers. Macromolecules.

[57] Lee H., Boyce J.R., Nese A., Sheiko S.S., Matyjaszewski K. (2008). pH-induced conformational changes of loosely grafted molecular brushes containing poly(acrylic acid) side chains. Polymer.

[58] Grube M., Leiske M.N., Schubert U.S., Nischang I. (2018). POx as an alternative to PEG? A hydrodynamic and light scattering study. Macromolecules.

[59] Gubarev A.S., Monnery B.D., Lezov A.A., Sedlacek O., Tsvetkov N.V., Hoogenboom R., Filippov S.K. (2018). Conformational properties of biocompatible poly(2-ethyl-2-oxazoline)s in phosphate buffered saline. Polym. Chem..

[60] Tsvetkov V.N. (1989). Rigid-Chain Polymers.

[61] Daoud M., Cotton J.P. (1982). Star shaped polymers: A model for the conformation and its concentration dependence. J. Phys. France.

[62] Baek J.B., Tan L.S. (2008). Synthesis and properties of polyetherketoneblock-polybenzobisthiazole-block-polyetherketone ABA triblock copolymers. Macromolecules.

[63] Gitsov I., Frechet J.M.J. (1993). Solution and solid—State properties of hybrid linear-dendritic block-copolymers. Macromolecules.

[64] Jeong M., Mackay M.E., Vestberg R., Hawker C.J. (2001). Intrinsic viscosity variation in different solvents for dendrimers and their hybrid copolymers with linear polymers. Macromolecules.

[65] Passeno L.M., Mackay E.M., Baker L.G. (2006). Conformational changes of linear-dendrimer diblock copolymers in dilute solution. Macromolecules.

[66] Zakharova O.G., Simonova M.A., Tarasova E.V., Filippov A.P., Semchikov Y.D. (2009). Model and hybrid polystyrenes containing trispentafluorophenylgermyl end groups. Int. J. Polym. Anal. Charact..

[67] Sung J.H., Lee D.C. (2001). Molecular shape of poly(2-ethyl-2-oxazoline) chains in THF. Polymer.

[68] Hoogenboom R. (2007). Poly(2-oxazoline)s: Alive and kicking. Macromol. Chem. Phys..

[69] Ivanova R., Komenda T., Bonne T.B., Lüdtke K., Mortensen K., Pranzas P.K., Jordan R., Papadakis C.M. (2008). Micellar Structures of Hydrophilic/Lipophilic and Hydrophilic/Fluorophilic Poly(2-oxazoline) Diblock Copolymers in Water. Macromol. Chem. Phys..

[70] Obeid R., Maltseva E., Thünemann A.F., Tanaka F., Winnik F.M. (2009). Temperature response of self-assembled micelles of telechelic hydrophobically modified poly(2-alkyl-2-oxazoline)s in water. Macromolecules.

[71] Trinh L.T.T., Lambermont-Thijs H.M.L., Schubert U.S., Hoogenboom R., Kjøniksen A.-L. (2012). Thermoresponsive poly(2-oxazoline) block copolymers exhibiting two cloud points: Complex multistep assembly behavior. Macromolecules.

[72] Takahashi R., Sato T., Terao K., Qiu X.-P.,  Winnik F.M. (2012). Self-Association of a Thermosensitive Poly(alkyl-2-oxazoline) Block Copolymer in Aqueous Solution. Macromolecules.

[73] Trzebicka B., Koseva N., Mitova V., Dworak A. (2010). Organization of poly(2-ethyl-2-oxazoline)-block-poly(2-phenyl-2-oxazoline) copolymers in water solution. Polymer.

[74] Schartl W. (2007). Light Scattering from Polymer Solutions and Nanoparticle Dispersions.

[75] Cagli E., Yildirim E., Yang S.-W., Erel-Goktepe I. (2019). An Experimental and Computational Approach to pH-Dependent Self-Aggregation of Poly(2-isopropyl-2-oxazoline). J. Polym. Sci. Part B Polym. Phys..

[76] Dworak A., Trzebicka B., Kowalczuk A., Tsvetanov C., Rangelov S. (2014). Polyoxazolines—mechanism of synthesis and solution properties. Polimery.

[77] Boerman M.A., Van der Laan H.L., Bender J.C.M.E., Hoogenboom R., Jansen J.A., Leeuwenburgh S.C., Van Hest J.C.M. (2016). Synthesis of pH- and Thermoresponsive Poly(2-n-propyl-2-Oxazoline) Based Copolymers. J. Polym. Sci. Part A Polym. Chem..

[78] Bivigou-Koumba A.M., Görnitz E., Laschewsky A., Müller-Buschbaum P., Papadakis C.M. (2010). Thermoresponsive amphiphilic symmetrical triblock copolymers with a hydrophilic middle block made of poly(N-isopropylacrylamide): Synthesis, self-organization, and hydrogel formation. Colloid Polym. Sci..

[79] Tarabukina E.B., Simonova M.A., Bucatariu S., Harabagiu V., Fundueanu G., Filippov A.P. (2016). Behavior of Thermo- and pH-responsive Copolymer of N-Isopropylacrylamide and Maleic Acid in Aqueous Solution. Int. J. Polym. Anal. Char..

[80] Aseyev V., Tenhu H., Winnik F.M. (2011). Non-ionic Thermoresponsive Polymers in Water. Adv. Polym. Sci..

[81] Uyama H., Kobayashi S. (1992). A Novel Thermo-Sensitive Polymer. Poly(2-*iso*-propyl-2-oxazoline). Chem. Lett..

[82] Meyer M., Antonietti M., Schlaad H. (2007). Unexpected thermal characteristics of aqueous solutions of poly(2-isopropyl-2-oxazoline). Soft Matter.

[83] Saha A., Ramakrishnan S. (2008). AB2 + A type copolymerization approach for the preparation of thermosensitive PEGylated Hyperbranched polymers. Macromolecules.

[84] Contreras M.M., Mattea C., Rueda J.C., Stapf S., Bajd F. (2015). Synthesis and characterization of block copolymers from 2-oxazolines. Des. Monomers Polym..

[85] Gao Y., Wong K.Y., Ahiabu A., Serpe M.J. (2016). Sequential and controlled release of small molecules from poly(N-isopropylacrylamide) microgel-based reservoir devices. J. Mater. Chem. B.

[86] Amirova A., Rodchenko S., Filippov A. (2016). Time dependence of the aggregation of star-shaped poly(2-isopropyl-2-oxazolines) in aqueous solutions. J. Polym. Res..

[87] Filippov A.P., Tarabukina E.B., Simonova M.A., Kirila T.U., Fundueanu G., Harabagiu V., Constantin M., Popescu I. (2015). Synthesis and investigation of double stimuli-responsive behavior of N-isopropylacrylamide and maleic acid copolymer. J. Macromol. Sci. Part B Phys..

