# Supplementary material for: Synthesis, Structure, Hydrodynamics and Thermoresponsiveness of Graft Copolymer with Aromatic Polyester Backbone at Poly(2-isopropyl-2-oxazoline) Side Chains
Source: Polymers (Basel). 2020 Nov 10;12(11):2643. doi: 10.3390/polym12112643 (PMC7698206; doi:10.3390/polym12112643)
Supplement: Supplementary file 1 [file polymers-12-02643-s001.pdf]

Supplemental information

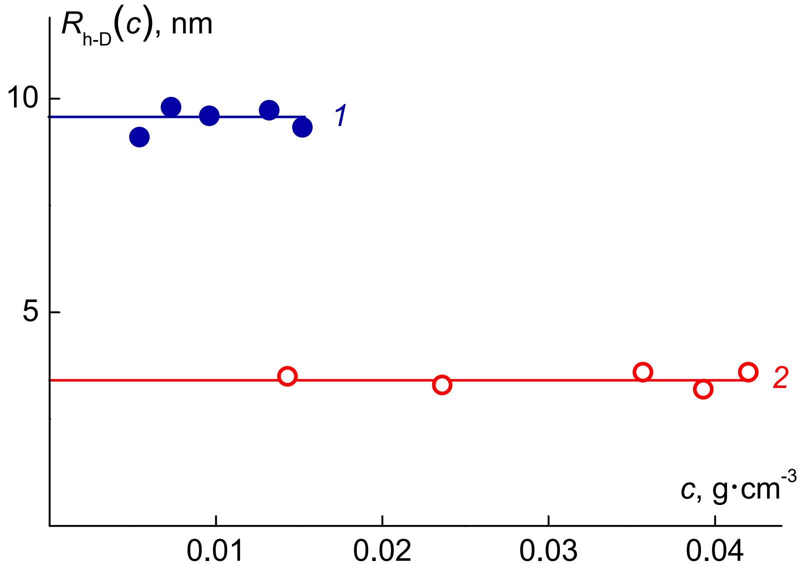

Figure S1. The concentration dependences of hydrodynamic radius $R_{h-D}(c)$ for solutions of APE-g-PiPrOx (1) and PiPrOx-m (2) in nitropropane.

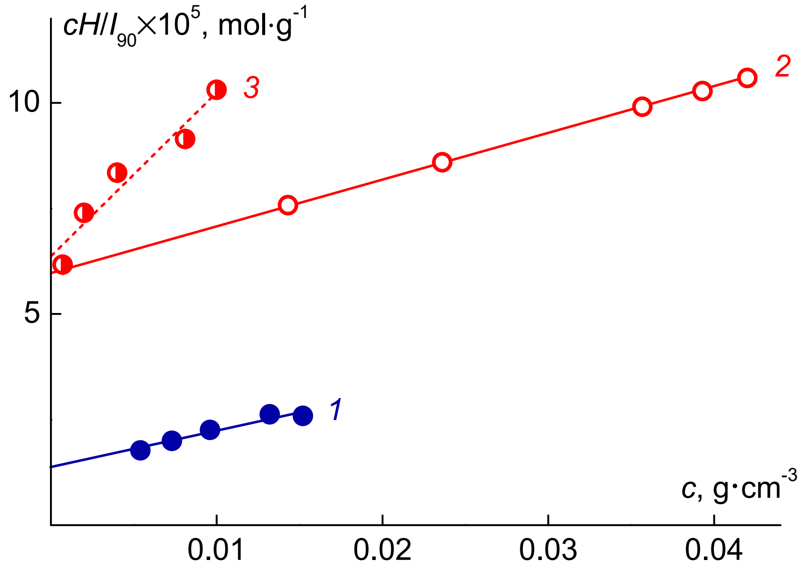

Figure S2. The concentration dependences of light scattering intensity I for solutions of APE-g-PiPrOx (1) and PiPrOx-m (2) in nitropropane and PiPrOx-m in water (3).

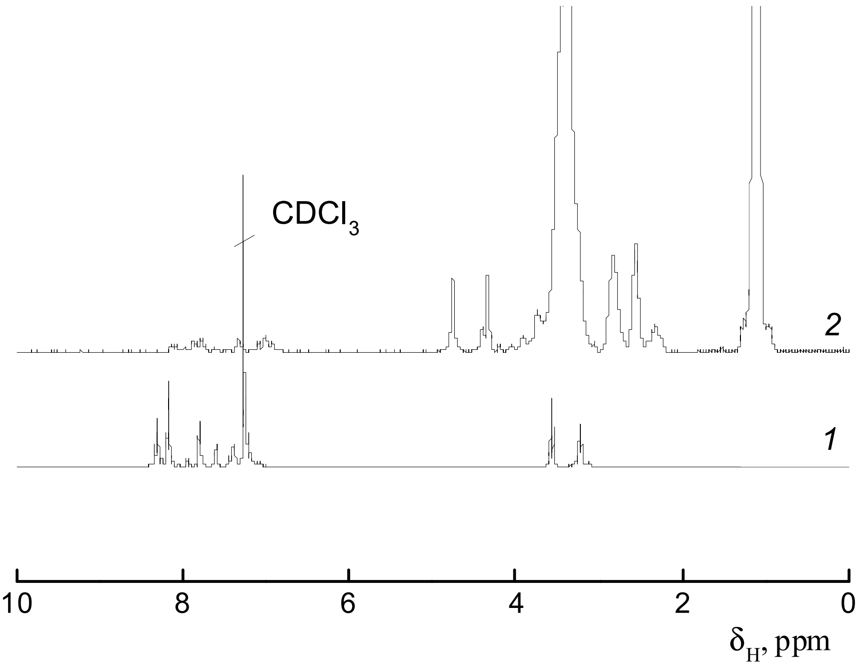

Figure S3. NMR spectra of APE macroinitiator (1) and graft copolymer APE-graft-PiPrOx.

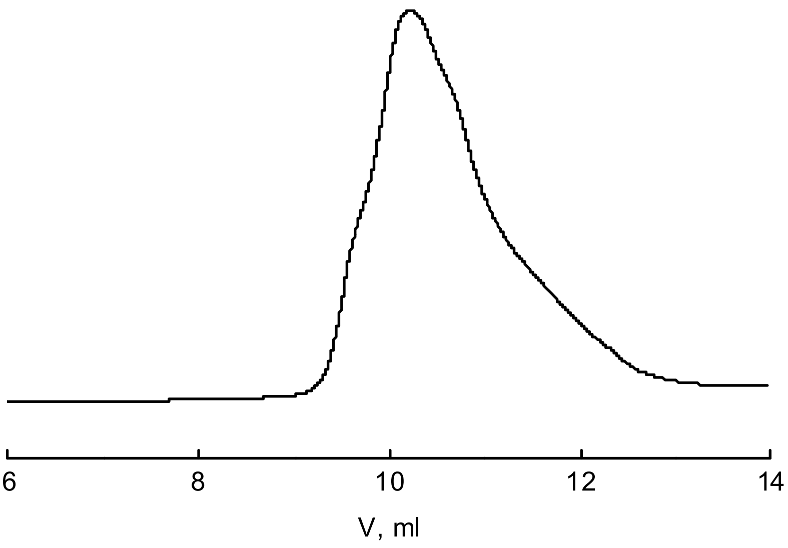

Figure S4. GPC trace of APE-graft-PiPrOx.

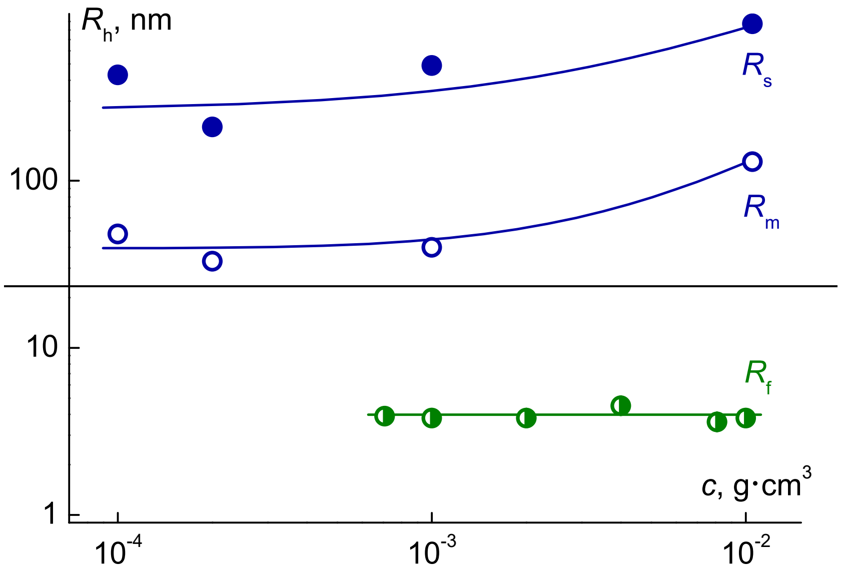

Figure S5. The concentration dependences of hydrodynamic radii R_m and R_s for aqueous solutions of APE-g-PiPrOx and R_h for aqueous solutions of PiPrOx-m at low temperatures.

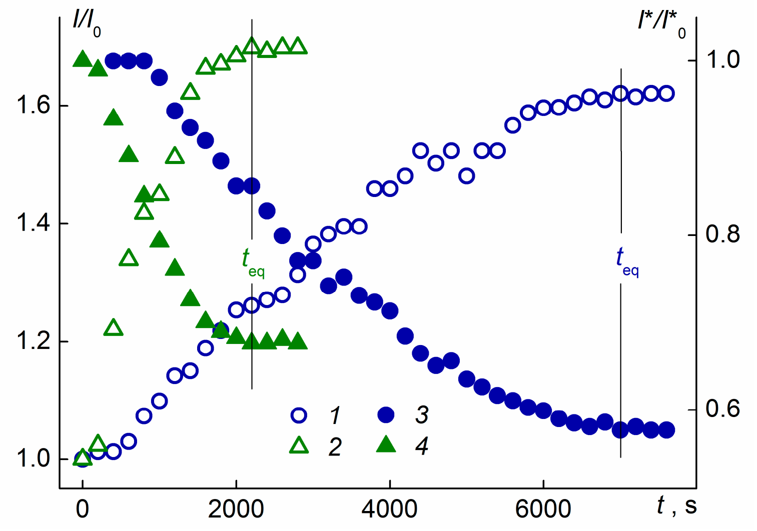

Figure S6. Dependences of light scattering intensity I/I_0 (*1*, *2*) and optical transmission I^*/I^*_0 (*3*, *4*) on time t for APE-g-PiPrOx solutions at c = 0.00010 g cm^{-3} (*1*, *3*) and for PiPrOx-m solutions at c = 0.0040 g cm^{-3} (*2*, *4*). I_0 and I^*_0 are the intensity of light scattering and optical transmission at t = 0, respectively.

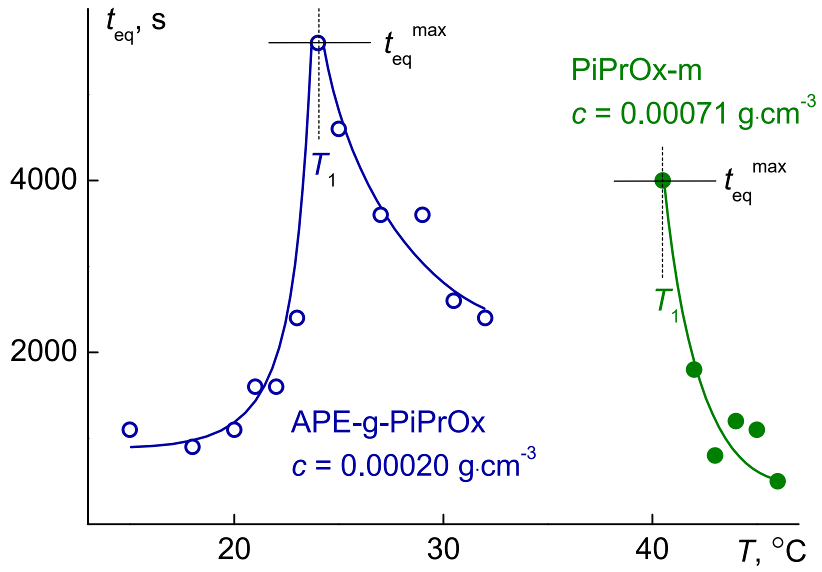

Figure S7. Temperature dependences of t_{eq} for APE-g-PiPrOx and PiPrOx-m.
